# VFFVA: dynamic load balancing enables large-scale flux variability analysis

**DOI:** 10.1186/s12859-020-03711-2

**Published:** 2020-09-29

**Authors:** Marouen Ben Guebila

**Affiliations:** Department of Biostatistics, Harvard T.H. Chan School of Public Health, Boston, MA, USA

**Keywords:** Metabolic models, Flux variability analysis, High performance computing, Systems biology

## Abstract

**Background:**

Genome-scale metabolic models are increasingly employed to predict the phenotype of various biological systems pertaining to healthcare and bioengineering. To characterize the full metabolic spectrum of such systems, Fast Flux Variability Analysis (FFVA) is commonly used in parallel with static load balancing. This approach assigns to each core an equal number of biochemical reactions without consideration of their solution complexity.

**Results:**

Here, we present Very Fast Flux Variability Analysis (VFFVA) as a parallel implementation that dynamically balances the computation load between the cores in runtime which guarantees equal convergence time between them. VFFVA allowed to gain a threefold speedup factor with coupled models and up to 100 with ill-conditioned models along with a 14-fold decrease in memory usage.

**Conclusions:**

VFFVA exploits the parallel capabilities of modern machines to enable biological insights through optimizing systems biology modeling. VFFVA is available in C, MATLAB, and Python at https://github.com/marouenbg/VFFVA.

## Background

Constraint-based reconstruction and analysis (COBRA) methods enable the study of metabolic pathways in bacterial [1] and human [2] systems, in time and space [3]. The metabolic models are usually formulated as linear systems [4] that are often under-determined [5], therefore several solutions could satisfy the subjected constraints. The set of alternate optimal solutions (AOS) describes the range of reaction rates that achieve the optimal objective such as biomass production. The AOS space is quantified using flux variability analysis (FVA) [5], which provides a range of minimum and maximum values for each variable of the system. FVA has been applied to find blocked reactions in the network [6], quantify the fitness of macrophages after the infection of *Mycobacterium tuberculosis* [7], resolve thermodynamically infeasible loops [8], and compute the essentiality of reactions [9].

fastFVA (FFVA) [10], a recent implementation of FVA allowed to gain substantial speed over the fluxvariability COBRA toolbox MATLAB function [11]. Two main elements were decisive in the improvement: First, the C implementation of FFVA was more flexible in comparison to MATLAB [12], allowing the use of the CPLEX C API. The second was the use of the same LP object, which avoided solving the program from scratch in every iteration, thereby saving presolve time. FFVA is compiled as MATLAB Executable (MEX) file, that can be called from MATLAB directly.

However, given the growing size of metabolic models, FFVA is usually run in parallel. Parallelism simply relies on allocating the cores through MATLAB parpool function [12] and running the iterations through parfor loop. The load is statically balanced over the workers such as they process an equal amount of iterations. Nevertheless, LPs vary in complexity and their solution time varies greatly. Therefore, the static load balancing setting does not guarantee an equal processing time among the workers. For example, the workers that were assigned a set of fast-solving LPs process their chunk of iterations and stay idle, waiting to synchronize with the remaining slower workers, which can result in larger run times globally. These situations can be inherent to the model such as Metabolism and Expression (ME) coupled models [13] that can be ill-conditioned. Also, intractable objective functions can induce an imbalance in the parallel distribution of metabolic reactions such as the generation of warmup points for sampling. Here we present veryfastFVA (VFFVA), which is a standalone C implementation of FVA, that has a lower level management of parallelism over FFVA. The significant contribution is the management of parallelism through a hybrid integration of parallel libraries OpenMP [14] and MPI [15], for shared memory and non-shared memory systems respectively. While keeping the up-mentioned advantages of FFVA, load balancing in VFFVA was scheduled dynamically to guarantee equal run times between the workers. The input does not rely on MATLAB anymore as the LP is read in the standard.*mps* file, that can be obtained from.*mat* files through a provided converter. The improvements in the implementation allowed to speed up the analysis by a factor of three and up to 100 with ill-conditioned problems and reduced memory requirements 14-fold in comparison to FFVA and the Julia-based distributedFBA implementation [16].

Taken together, as metabolic models are steadily growing in number and complexity, their analysis requires the design of efficient tools. VFFVA allows exploiting the multi-core specifications of modern machines to run more simulations in less time thereby enabling biological discovery.

## Implementation

### Flux variability analysis

The metabolic model of a biological system is formulated as an LP problem that has *n* variables (reactions) bounded by lower bound *lb*_(*n*,1)_ and upper bound *ub*_(*n*,1)_ vectors. The matrix *S*_(*m*,*n*)_ represents the stoichiometric coefficients of each of the *m* metabolites involved in the *n* reactions. The system is usually considered in its steady-state and is constrained by *S*.*v*=0, which is also referred to as Flux Balance Analysis (FBA) [17]. An initial LP optimizes for the objective function of the system to obtain a unique optimum, e.g., biomass maximization, like the following:

1 $$  \begin{array}{ll@{}ll} \text{maximize} & \ Z_{biomass}=c^{T}_{biomass}v &\\ \text{subject to}\\ & S.v=0 \\ & lb< v< ub \end{array}  $$

The system being under-determined (*m*<*n*), there can be an infinity of solution vectors *v*_(*n*,1)_ that satisfy the unique optimal objective (*c*^*T*^*v*), with *c*_(*n*,1)_ as the objective coefficient vector. In a second step, in order to delineate the AOS space, the objective function is set to its optimal value followed by an iteration over the *n* dimensions of the problem. Consequently, each of the reactions is set as a new objective function to maximize (minimize) and obtain the maximal (minimal) value of the reaction range. The total number of LPs is then equal to 2*n* in the second step which is described as the following:

2 $$  \begin{array}{ll@{}ll@{}ll} \text{For each reaction} & i \in \ [1,n] &\\ & \text{set} & \ c_{i}=1 &\\ & \text{max/min} & \ Z_{i}=c^{T}v &\\ & \text{subject to}\\ & & S.v=0 \\ & & c^{T}_{biomass}v=Z_{biomass} \\ & & lb< v< ub \end{array}  $$

The obtained minimum and maximum objective values for each dimension define the range of optimal solutions.

### Management of parallelism

Problem 2 is entirely parallelizable through distributing the 2*n* LPs among the available workers. The strategy used so far in the existing implementations was to divide 2*n* equally among the workers. Nevertheless, the solution time can vary widely between LPs because ill-conditioned LPs can induce numerical instabilities requiring longer solution times. Consequently, dividing equally the LPs among the workers does not ensure an equal load on each worker.

Since it is challenging to estimate a priori the run time of an LP, the load has to be dynamically balanced during the execution of the program.

In shared memory systems, Open Multi-Processing (OpenMP) library allows balancing the load among the threads dynamically such that every instruction runs for an equal amount of time. The load is adjusted dynamically, depending on the chunks of the problem processed by every thread. At the beginning of the process, the scheduler will divide the original problem in chunks and will assign the workers a chunk of iterations to process. Each worker that completes the assigned chunk will receive a new one until all the LPs are processed.

In systems that do not share memory, Message Passing Interface (MPI) was used to create instances of Problem 2. Every process then calls the shared memory execution through OpenMP.

In the end, the final program is comprised of a hybrid MPI/OpenMP implementation of parallelism which allows great flexibility of usage, particularly in High-Performance Computing (HPC) setting.

### Another application: generation of warmup points

The uniform sampling of metabolic models is a common unbiased tool to characterize the solution space and determine the flux distribution per reaction [18, 19]. Sampling starts from pre-computed solutions called warmup points, where the sampling chains start exploring the solution space. The generation of *p*≥2*n* warmup points is done similarly to FVA. The first 2*n* points are solutions of the FVA problem, while the points ≥2*n* are solutions corresponding to a randomly generated coefficient vector *c*. The optimization of a randomly generated objective function can be a source of imbalance in the parallel distribution of load in FVA, which makes this application particularly interesting in dynamic load balancing. Another difference with FVA lies in the storage of the solutions *v* rather than the optimal objective *c*^*T*^*v*. The generation of 30,000 warmup points was compared using the COBRA toolbox function createWarmup_MATLAB_ and a dynamically load-balanced C implementation createWarmup_VF_ that was based on VFFVA.

### Model description

FFVA and VFFVA were tested on a selection of models [10]. The models (Table 1) are characterized by the dimensions of the stoichiometric matrix *S*_*m*,*n*_. Each model represents the metabolism of human or bacterial systems. Models pertaining to the same biological system with different *S* matrix size, have different levels of granularity and biological complexity. The exchange reactions were set to the default values specified in the model. E_Matrix and E_c__Matrix are ME models depicting metabolism and expression, while all the others are metabolism only models.

**Table 1:** Model size and description

Model	Organism	Size
Ecoli_core [20]	*Escherichia coli*	(72,95)
P_putida [21]	*Pseudomonas putida*	(911,1060)
EcoliK12 [22]	*Escherichia coli*	(1668,2382)
Recon2 [23]	*Homo sapiens*	(4036,7324)
E_Matrix [24]	*Escherichia coli*	(11991,13694)
E_c__Matrix [25]	*Escherichia coli*	(13047,13726)
WBM [2]	*Homo sapiens*	(157056,80016)

### Hardware and software

VFFVA and createWarmup_VF_ were run on a Dell HPC machine with 72 Intel Xeon E5 2.3 GHz cores and 768 GB of memory. The current implementation was tested with Open MPI v1.10.3, OpenMP 3.1, GCC 4.7.3, and IBM ILOG CPLEX academic version (12.6.3). FFVA and createWarmup_MATLAB_ were tested with MATLAB 2014b [12] and distributedFBA was run on Julia v0.5. ILOG CPLEX was called with the following parameters:

$$ \begin{array}{ll@{}ll} \texttt{PARALLELMODE=1} &\\ \texttt{THREADS=1} &\\ \texttt{AUXROOTTHREADS=2} &\\ \end{array} $$

Additionally, coupled models with scaling infeasibilities might require turning off the scaling:

$$ \begin{array}{ll@{}ll} \texttt{SCAIND=-1} &\\ \end{array} $$

The call to VFFVA is done from bash as follows:

mpirun -np <nproc> --bind-to none -x OMP_NUM_THREADS=<nthr> veryfastFVA <model.mps> <optPerc> <scaling> <rxns>

where nproc is the number of non-shared memory processes, *nthr* is the number of shared memory threads, *optPerc* is the percentage of the optimal objective of the metabolic model considered for the analysis, *scaling* is CPLEX scaling parameter where 0 leaves it to the default (equilibration) and -1 sets it to unscaling such as for coupled models, and *rxns* is an optional user-defined subset of reactions to analyze. createWarmup_VF_ was called in a similar fashion:

mpirun -np <nproc> --bind-to none -x OMP_NUM_THREADS=<nthr> createWarmupPts <model.mps> <scaling>

For large models, OpenMP threads were bound to physical cores through setting the environment variable

$$ \begin{array}{ll@{}ll} \texttt{OMP\_PROC\_BIND=TRUE} &\\ \end{array} $$

while for small models, setting the variable to FALSE yielded faster run times. The schedule is set through the environment variable

$$ \begin{array}{ll@{}ll} \texttt{OMP\_SCHEDULE=schedule,chunk} &\\ \end{array} $$

where schedule can be static, dynamic or guided, and chunk is the minimal number of iterations processed per worker at a time.

### Other possible implementations

The presented software can be implemented in Fortran since the library OpenMP is supported as well. Additionally, Python’s multiprocessing library allows to balance the load dynamically between non-shared memory processes, but the parallelism inside one process is often limited to one thread by the Global Interpreter Lock (GIL). This limitation could be circumvented through using OpenMP and Cython [26]. The unique advantage of the presented implementation of VFFVA is the deployment of two levels of parallelism following a hierarchical model where MPI processes are at a top-level and OpenMP threads at a lower level. The MPI processes manage the coarse-grained parallelism, and OpenMP threads manage the finer-grained tasks that share memory and avoid copying the original problem, which increases performance and saves consequent memory. This architecture adapts seamlessly with modern distributed hardware in HPC setting. MATLAB and Python wrappers of the C code were provided at https://github.com/marouenbg/VFFVA.

## Results

The OpenMP/MPI hybrid implementation of VFFVA allowed to gain a significant speedup over the static load balancing approach. In this section, the run times of VFFVA were compared to FFVA at different settings followed by a comparison of the different strategies of load balancing with respect to their impact on the run time per worker. In contrast to previous work where FFVA was benchmarked in serial runs [10], in the present work the emphasis was put upon parallel run times.

### Parallel construct in a hybrid openMP/MPI setting

**Fig. 1:**
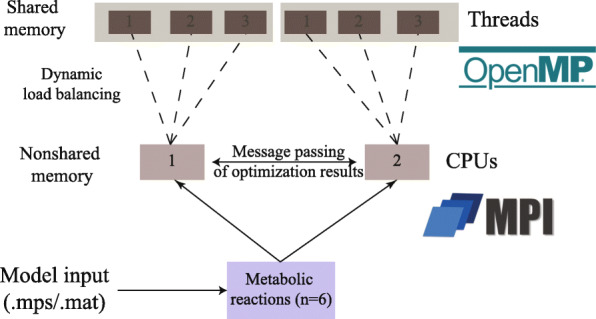
Hybrid OpenMP/MPI implementation of FVA ensures two levels of parallelism. The distribution of tasks is implemented following a hierarchical model where MPI manages coarse-grained parallelism in non-shared memory systems. At a lower level, OpenMP processes within each MPI process manage fine-grained parallelism taking advantage of the shared memory to improve performance

The MATLAB implementation of parallelism through the parallel computing toolbox provides great ease-of-use, wherein two commands only are required to allocate and launch parallel jobs. Also, it saves the user the burden of finding out whether the jobs are run on shared or non-shared systems. VFFVA provides the user with a similar level of flexibility as it supports both types of systems while guaranteeing the same numerical results as FVA in double precision (Figure S1). Besides, it allows accessing advanced features of OpenMP and MPI such as dynamic load balancing. The algorithm starts first by assigning chunks of iterations to every CPU (Fig. 1), where a user-defined number of threads simultaneously process the iterations. In the end, the CPUs synchronize and pass the result vector to the main core to reduce them to the final vector.

The main contributions of VFFVA are the complete use of C, which impacted mainly the computing time of small models (*n*<3000), and the dynamic load balancing that was the main speedup factor for larger models.

### Impact on computing small models

VFFVA and FFVA were run five times on small models, i.e., Ecoli_core, EcoliK12, and P_putida. VFFVA had at least 20-fold speedup on the average of the five runs (Table 2). The main contributing factor was the use of C over MATLAB in all steps of the analysis. In particular, the loading time of MATLAB Java machine and the assignment of workers through parpool was much greater than the analysis time itself.

**Table 2:** Comparison of run times of FFVA and VFFVA in small models in seconds. The results are presented as the mean and standard deviation of five runs

Model	FFVA mean(std) loading and analysis time	VFFVA mean(std) loading and analysis time	FFVA mean(std) analysis only
2 cores			
Ecoli_core	19.5(0.5)	0.2(0.01)	0.37(0.1)
P_putida	19.2(0.7)	0.6(0.02)	0.81(0.09)
EcoliK12	20.4(0.6)	2.2(0.06)	2.41(0.09)
4 cores			
Ecoli_core	19.6(0.6)	0.2(0.005)	0.32(0.01)
P_putida	19.4(1)	0.5(0.02)	0.61(0.01)
EcoliK12	20(0.8)	1.3(0.04)	1.64(0.08)
8 cores			
Ecoli_core	19.4(0.5)	0.2(0.03)	0.35(0.05)
P_putida	19.6(0.7)	0.4(0.04)	0.53(0.009)
EcoliK12	20(0.49)	0.9(0.01)	1.22(0.08)
16 cores			
Ecoli_core	20.2(0.4)	0.2(0.008)	0.41(0.05)
P_putida	19.5(0.4)	0.4(0.04)	0.51(0.03)
EcoliK12	22(0.7)	0.7(0.01)	0.87(0.03)
32 cores			
Ecoli_core	22.2(0.4)	0.3(0.008)	0.6(0.12)
P_putida	21.5(0.6)	0.4(0.01)	0.53(0.004)
EcoliK12	21.5(0.6)	0.6(0.03)	0.78(0.04)

The result highlighted the power of C in gaining computing speed, through managing the different low-level aspects of memory allocation and variable declaration.

In the analysis of large models, where MATLAB loading time becomes less significant, dynamic load balancing becomes the main driving factor of run time decrease.

### Impact on computing large models

**Fig. 2:**
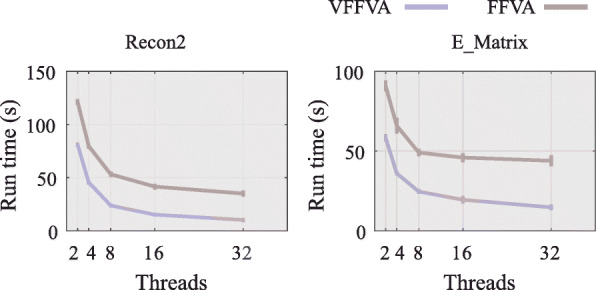
Run times of Recon2 and E_Matrix model using FFVA and VFFVA on 2,4,8,16, and 32 threads. The guided schedule was used in the benchmarking. The run time accounted for the creation of the parallel pool (loading time) and analysis time

The speedup gained on computing large models (Recon2 and E_Matrix) reached three folds with VFFVA (Fig. 2) at 32 threads with Recon 2 (35.17*s* vs 10.3*s*) and E_Matrix (44*s* vs 14.7*s*) for the loading and analysis time. In fact, with dynamic load balancing, VFFVA allowed to update the assigned chunks of iterations to every worker dynamically, which guarantees an equal distribution of the load. In this case, the workers that get fast-solving LPs, will get a larger number of iterations assigned. Conversely, the workers that get ill-conditioned LPs, e.g., having an *S* matrix with a large condition number, require more time to solve them and will get fewer LPs in total. Finally, all the workers synchronize at the same time to reduce the results. Particularly, the speedup achieved with VFFVA increased with the size of the models and the number of threads (Fig. 2E_Matrix). Finally, the different load balancing strategies (static, guided, and dynamic) were compared further with two of the largest models (Whole Body Model (WBM) and E_c__Matrix).

### Load management

Load management describes the different approaches to assign iterations to the workers. It can be static, where an even number of iterations is assigned to each worker. Guided schedule refers to dividing the iterations in chunks of size 2*n*/*workers* initially, with *n* equal to the number of reactions in the model, and *q*/*workers* afterward, with *q* equal to the remaining reactions after the initial assignment. The main difference with static balancing was the dynamic assignment of chunks, in a way that fast workers can process more iteration blocks. Finally, the is very similar to guided except that chunk size is given by the user, which allows greater flexibility. In the following section, the load balancing strategies of E_c__Matrix which is an ME coupled model and WBM were compared for the time required to load and perform the analysis.

#### Static schedule

**Fig. 3:**
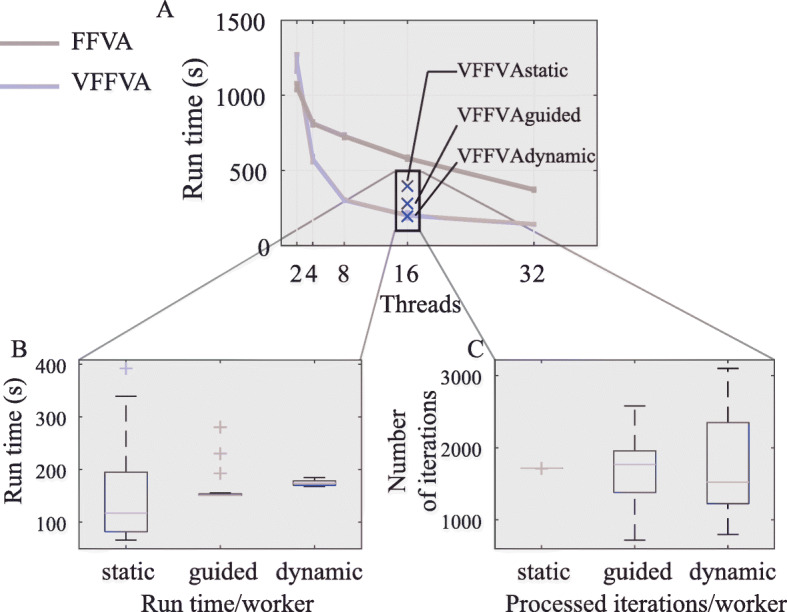
Run times of E_c__Matrix model. **a**-Run times of E_c__Matrix model at 2,4,8,16, and 32 threads using FFVA and VFFVA. The run time accounted for the creation of the parallel pool (loading time) and analysis time. **b**-Run time per worker in the static, guided, and using 16 threads. **c**-The number of iterations processed per worker in the static, guided, and using 16 threads

Using static schedule, VFFVA assigned an equal number of iterations to every worker. With 16 threads, the number of iterations per worker equaled 1715 and 1716 (Fig. 3c). Expectedly, the run time varied widely between workers (Fig. 3b) and resulted in a final time of 393*s*.

#### Guided schedule

With guided schedule (Fig. 3a), the highest speedup (2.9) was achieved with 16 threads (Fig. 3b). The iterations processed varied between 719 and 2581 and the run time per worker was quite comparable with a final run time equal to 281*s*.

#### Dynamic schedule

Using dynamic load balancing with a chunk size of 50 resulted in similar performance to the guided schedule. The final run time equaled 197*s*, while FFVA took 581*s*. An optimal chunk size has to be small enough to ensure a frequent update on the workers’ load, and big enough to take advantage of the solution basis reuse in every worker. At a chunk size of one, i.e., each worker is assigned one iteration at a time, the final solution time equaled 272*s*. In fact, for a small chunk size, the worker is updated often with new pieces of iterations, loses the stored solution basis of the previous problem, and has to solve the LP from scratch which slows the overall process.

**Fig. 4:**
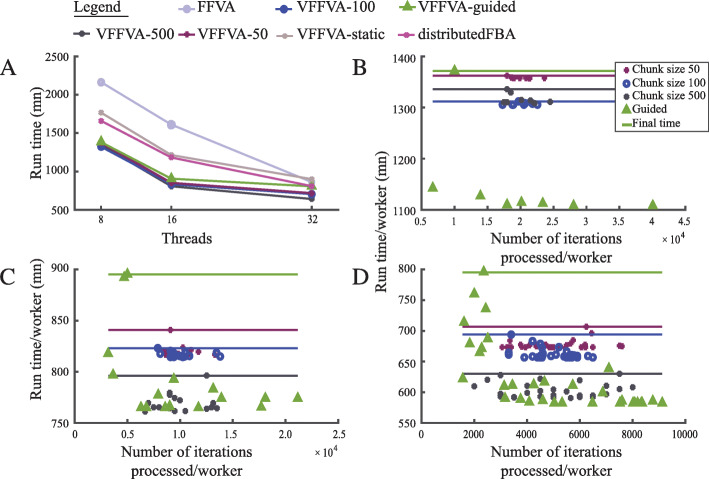
Run times per worker of WBM *Homo sapiens* metabolic model. **a**-Final run time of the different load balancing schedules at 8, 16, and 32 threads. The run time accounted for the creation of the parallel pool (loading time) and analysis time. **b**-Run time per worker as a function of the number of iterations processed using the guided schedule and the dynamic schedule with a chunk size of 50, 100, and 500 with eight threads, **c**-16 threads, and **d**-32 threads

Similarly, the computation of the solution space for WBM *Homo sapiens* metabolic model [2] (Fig. 4a) had a twofold speedup with 16 threads using a chunk size of 50 (806 *mn*) compared to FFVA (1611*mn*). The run times with guided schedule (905*mn*) and dynamic schedule with chunk size 100 (850*mn*) and chunk size 500 (851*mn*) were less efficient due to the slower update rate leading to a variable analysis time per worker (Fig. 4b,c,d). VFFVA on eight threads (1323*mn* with chunk size 50) proved comparable to FFVA (1214*mn*) and distributedFBA (1182*mn*) on 16 threads, thereby saving computational resources and time.

### Impact on memory usage

**Fig. 5:**
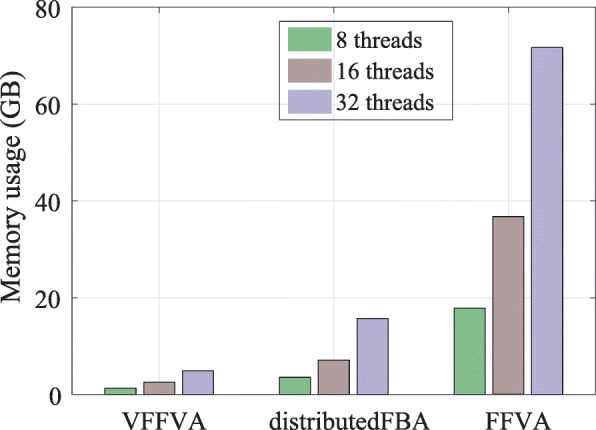
Physical memory usage at 8, 16, and 32 threads using FFVA, VFFVA, and distributedFBA highlights a lower memory usage with VFFVA

In MATLAB, the execution of *j* parallel jobs implies launching *j* instances of MATLAB. On average, one instance needs 2 GB. In a parallel setting, the memory requirements are at a minimum 2*j* GB, which can limit the execution of highly parallel jobs. In the Julia-based distributedFBA, the overall memory requirement exceeded 15 GB at 32 cores. VFFVA requires only the memory necessary to load *j* instances of the input model, which corresponds to the MPI processes as the OpenMP threads save additional memory through sharing one instance of the model. The differences between the FFVA and VFFVA get more pronounced as the number of threads increases (Fig. 5), i.e., 13.5-fold using eight threads, 14.2-fold using 16 threads, and 14.7-fold using 32 threads.

Finally, VFFVA outran FFVA and distributedFBA both on execution time and memory requirements (Table 3). The advantage becomes important with larger models and a higher number of threads, which makes VFFVA particularly suited for analyzing large-scale metabolic models in HPC setting.

**Table 3:** Comparative summary of the features of each implementation

Feature	VFFVA	distributedFBA	FFVA	FVA
Speed	++++	+++	++	+
Memory	+++	++	+	+
Load balancing	Dynamic	Static	Static	Static

### Creation of warmup points for sampling

Sampling the solution space of metabolic models is an unbiased method that allows to characterize the space of metabolic phenotypes, as opposed to FBA that provides single solutions, and FVA that computes solution ranges. The uniform sampling of the solution space is a time-consuming process that starts with the generation of warmup points to determine the initial starting points for sampling. This step is formulated similarly to FVA and could be accelerated using dynamic load balancing. The generation of 30,000 warmup points were compared using the COBRA toolbox function createWarmup_MATLAB_ and a dynamically load-balanced C implementation createWarmup_VF_ on a set of models (Table 4). Since the COBRA toolbox implementation does not support parallelism, it was run on a single core and the run time was divided by the number of cores to obtain an optimistic approximation of the parallel run times. The speedup achieved varied between four up to a factor of 100 in the different models (Table 4). Similarly to FFVA [10], the main driving factor for the decrease in computation time was the C implementation that allowed to reuse the LP object in every iteration and to save presolve time. Equally, dynamic load balancing between the workers ensured a fast convergence time.

**Table 4:** Generation of sampling warmup points using dynamic load balancing

Model	createWarmup_MATLAB_	createWarmup_VF_					
Cores	1	1	2	4	8	16	32
Ecoli_core	149	2.8	1.8	0.8	0.7	0.5	0.5
P_putida	385	12.5	13	8	4	2	2
EcoliK12	801	49	43	23	10.4	9.5	9.1
Recon2	11346	288	186	30	32	24	21
E_Matrix	NA^*^	602	508	130	52	43	43
E_c__Matrix	NA^*^	5275	4986	924	224	118	117

^*^The generation of warmup points of E_Matrix and E_c__Matrix models did not converge after 20000*s*. The creation of warmup points can vary widely between runs as it involves the generation of a random *c* vector in the linear program. The runs were repeated three times and the average was reported.

The run times of the generation of 30,0000 warmup points for sampling of six metabolic models using the standard serial implementation createWarmup_MATLAB_ and the dynamic load balanced implementation createWarmup_VF_

In general, dynamic load balancing is a promising avenue for computing parallel FVA on ill-conditioned problems such as ME coupled models [13], the generation of warmup points, and loopless FVA [8, 27]. The sources of imbalance in metabolic models could be inherent to the model formulation like ME coupled models that represent processes at different scales. However, there were no correlation between the model condition number and the performance gain attributed to dynamic load balancing (Figure S2). A second cause of imbalance is the formulation of the objective function such as the case of the generation of warmup points, where the optimization of a randomly generated objective function induces a severe imbalance. In this case, dynamic load balancing was up to 100 times faster than static load balancing (Table 4) in particular with ME coupled models such as E_c__Matrix. This finding suggests that a combination of the ill-conditioning of the stoichiometric matrix and the formulation of the objective function could contribute to a large imbalance, therefore a larger benefit of using dynamic load balancing.

Taken together, the dynamic load balancing strategy allows efficient parallel solving of metabolic models through accelerating the computation of FVA and the fast preprocessing of sampling points thereby enabling the modeler to tackle large-scale metabolic models.

## Conclusions

Large-scale metabolic models of biological organisms are becoming widely used in the prediction of disease progression and the discovery of therapeutic targets [28]. The standard tools available in the modeler’s toolbox have to be up-scaled to meet the increasing demand in computational time and resources [29]. VFFVA is the precursor of the next generation of modeling tools that leverage the specifications of modern computers and computational facilities to enable biological insights through parallel and scalable systems biology analyses.

## Availability and requirements

Project name: VFFVAProject home page: https://github.com/marouenbg/VFFVAOperating system: Unix systemsProgramming language: C, MATLAB (>2014b), Python (>3.0)Other requirements: Open MPI (v1.10.3), OpenMP (v3.1), GCC (v4.7.3), IBM ILOG CPLEX free academic version (v12.6.3).License: MITAny restrictions to use by non-academics: None, conditional on a valid CPLEX license.

## Supplementary information

**Additional file 1** **Figure S1.** Comparison of VFFVA and FVA results in double precision using Ecoli_core metabolic model. **Figure S2.** Absence of the effect of model complexity on VFFVA speedup over FFVA.

## Data Availability

All the code and datasets are available in the Github repository https://github.com/marouenbg/VFFVAand as a code ocean capsule 10.24433/CO.1817960.v1.

## Abbreviations

AOS: Alternative solution space
COBRA: Constraint-based reconstruction and analysis
FBA: Flux balance analysis
FFVA: Fast flux variability analysis
FVA: Flux variability analysis
GIL: Global interpreter lock
GSMMs: Genome-scale metabolic models
HPC: High performance computing
LP: Linear program
MPI: Message passing interface
OpenMP: Open multi-processing
VFFVA: Very fast flux variability analysis
WBM: Whole body model
