# Supplementary material for: VFFVA: dynamic load balancing enables large-scale flux variability analysis
Source: BMC Bioinformatics. 2020 Sep 29;21:424. doi: 10.1186/s12859-020-03711-2 (PMC7523073; doi:10.1186/s12859-020-03711-2)
Supplement: Supplementary file 1 — Additional file 1 Figure S1. Comparison of VFFVA and FVA results in double precision using Ecoli_core metabolic model. Figure S2. Absence of the effect of model complexity on VFFVA speedup over FFVA. [file 12859_2020_3711_MOESM1_ESM.pdf]

Supplementary material to VFFVA: dynamic load balancing enables large-scale flux variability analysis

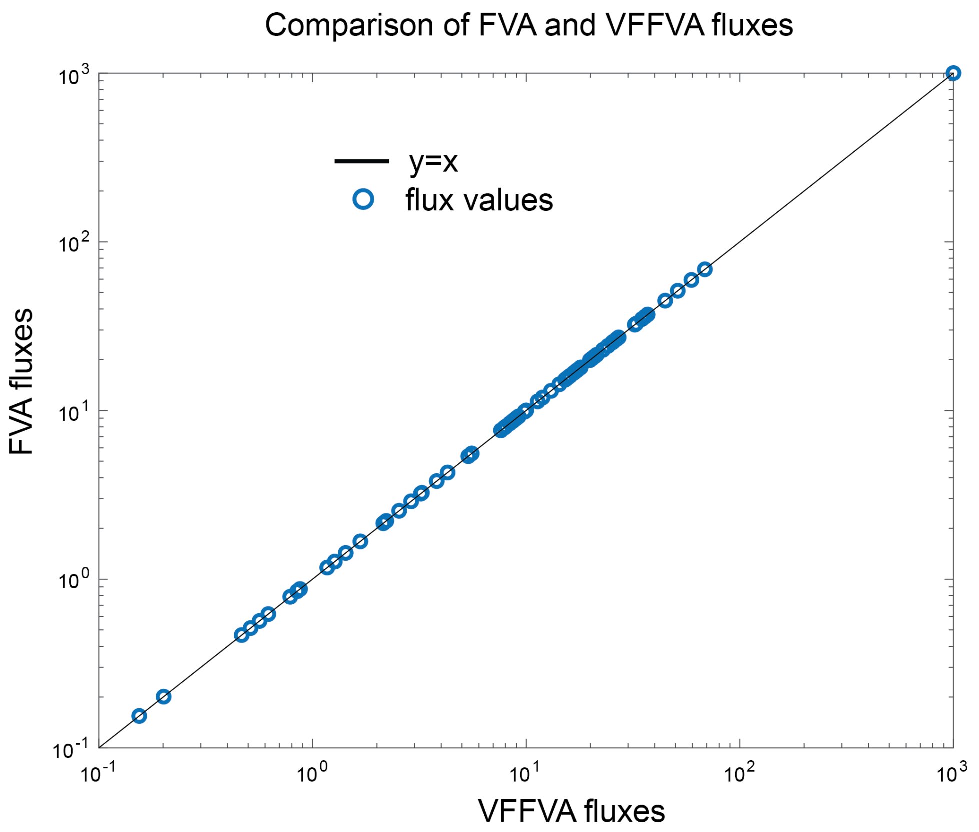

Figure S1: Comparison of VFFVA and FVA flux results in double precision using Ecoli_core metabolic model. The largest difference between the flux values is equal to $4.9314 * 10^{-7}$.

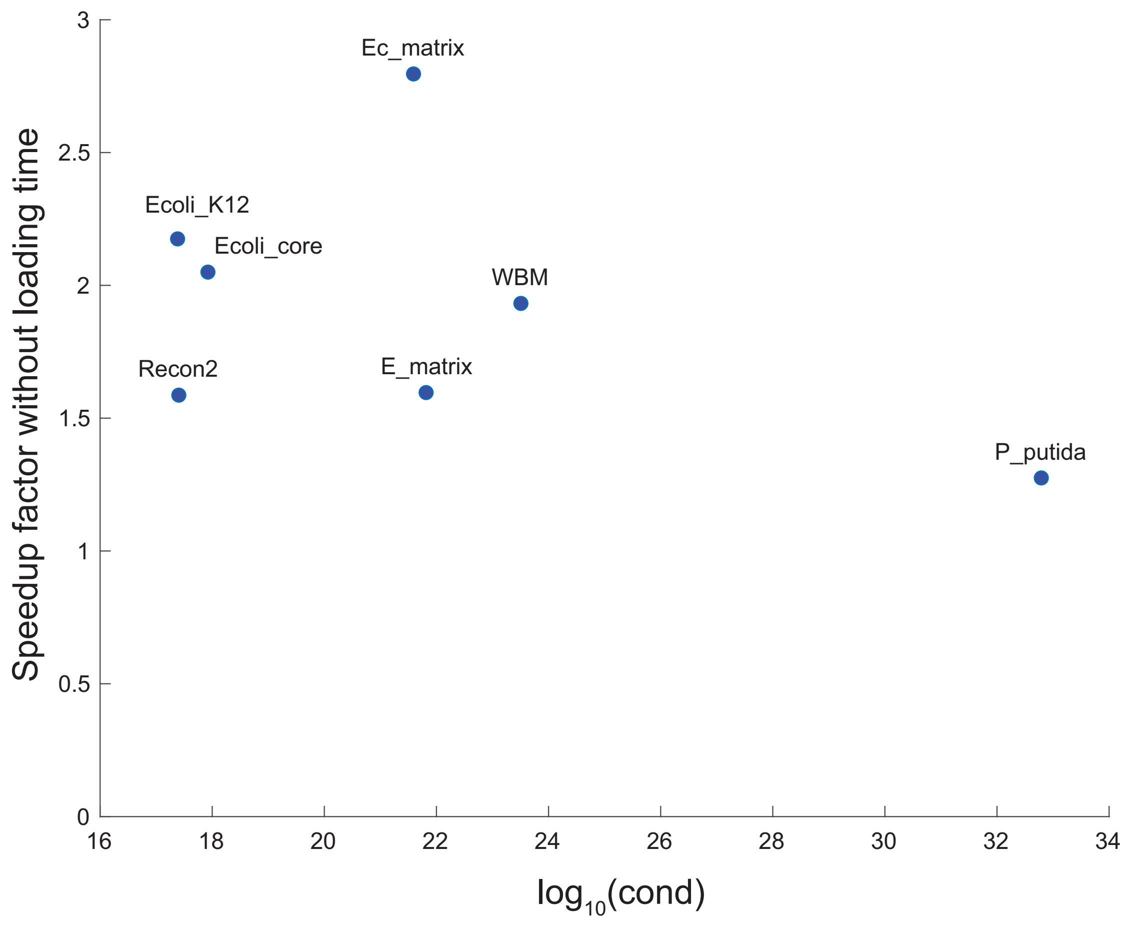

Figure S2: Absence of the effect of model complexity on VFFVA speedup over FFVA. The speedup of VFFVA over FFVA using 16 cores is represented as a function of model complexity expressed as the $\log_{10}$ of the condition number of the stoichiometric matrix S of the selected models. Only the fraction of speedup that is due to dynamic load balancing, i.e., excluding loading time, is considered in this analysis. $r = -0.45$, $p - value = 0.3$.
